# Spontaneous cervical and mediastinal hematoma due to rupture of inferior thyroid artery

**DOI:** 10.1016/j.radcr.2024.11.014

**Published:** 2024-11-30

**Authors:** Takayuki Yamada, Satoru Yanagaki, Nozomi Satani, Yuriko Kagaya, Tomoni Sato, Tomonori Matsuura, Teruyuki Sato, Naoya Noguchi, Nobuo Ohta

**Affiliations:** a Department of Radiology, Tohoku Medical and Pharmaceutical University, Sendai, Japan; b Department of Otolaryngology, Tohoku Medical and Pharmaceutical University, Sendai, Japan

**Keywords:** Spontaneous hematoma, Neck, Mediastinum, Thyroid artery, Rupture

## Abstract

A 62-year-old man was referred to our hospital presenting with a sore throat, dyspnea, and cervical swelling. Initial precontrast CT scans revealed a cervical and mediastinal hematoma, along with a hemothorax. Further dynamic contrast-enhanced CT scans indicated contrast media extravasation dorsal to the right thyroid gland lobe, suggesting a rupture of the right inferior thyroid artery or a parathyroid adenoma. Following endotracheal intubation, angiography confirmed extravasation from the right inferior thyroid artery. Transarterial embolization (TAE) was successfully performed using a gelatin sponge. The cervical and mediastinal hematoma were surgically excised, and the right inferior parathyroid gland was simultaneously resected. Pathological examination revealed no neoplastic components.

## Introduction

Spontaneous cervical hematoma is a rare condition with various underlying causes. It can lead to upper airway stenosis, a potentially life-threatening situation that often requires endotracheal intubation. Rapid identification of the cause is crucial for timely intervention. Similarly, spontaneous mediastinal hematoma is rare and shares some overlapping causes with cervical hematoma. In this report, we present a case of spontaneous cervical and mediastinal hematoma where the cause was promptly identified, leading to immediate and appropriate treatment by physicians.

## Case report

A 62-year-old man was admitted to our hospital presenting with sore throat and dyspnea. Nine days prior to admission, he experienced these symptoms, which worsened when lying supine. The day before his admission, he noticed swelling in his neck and sought care at another hospital. He was referred to our hospital the following day. His medical history included hypertension, diabetes mellitus, chronic renal failure, and cerebral infarction, and he had undergone hemodialysis. Upon admission, physical examination revealed swelling in the neck, pharynx, and larynx. Laboratory data indicated an elevated white blood cell count of 12,700/μL and a C-reactive protein (CRP) level of 23.48 mg/dL, suggesting an active inflammatory process.

**Fig. 1:**
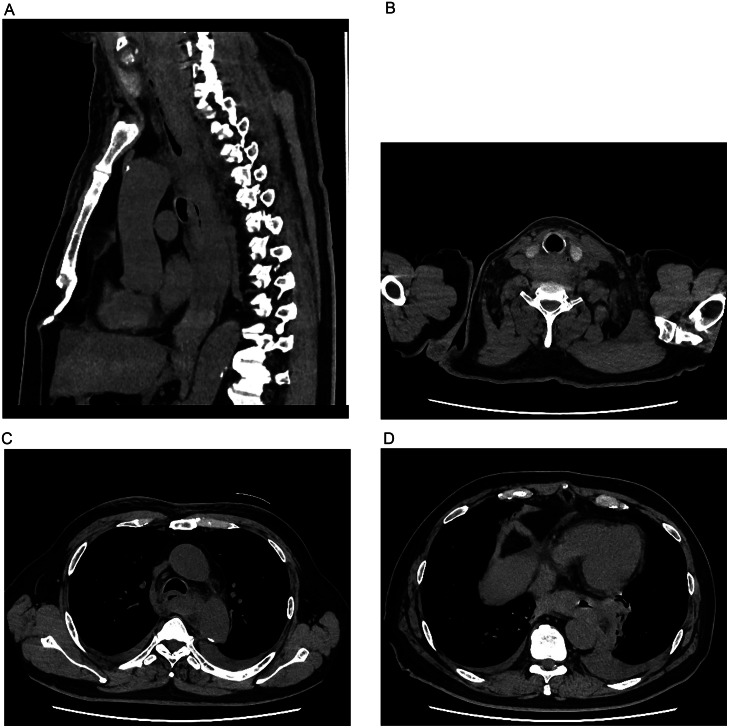
(A) Precontrast CT scans in the sagittal plane (A) and axial plane (B-D) depict an extensive hematoma extending from the neck to the mediastinum, surrounding the esophagus (C, D).

**Fig. 2:**
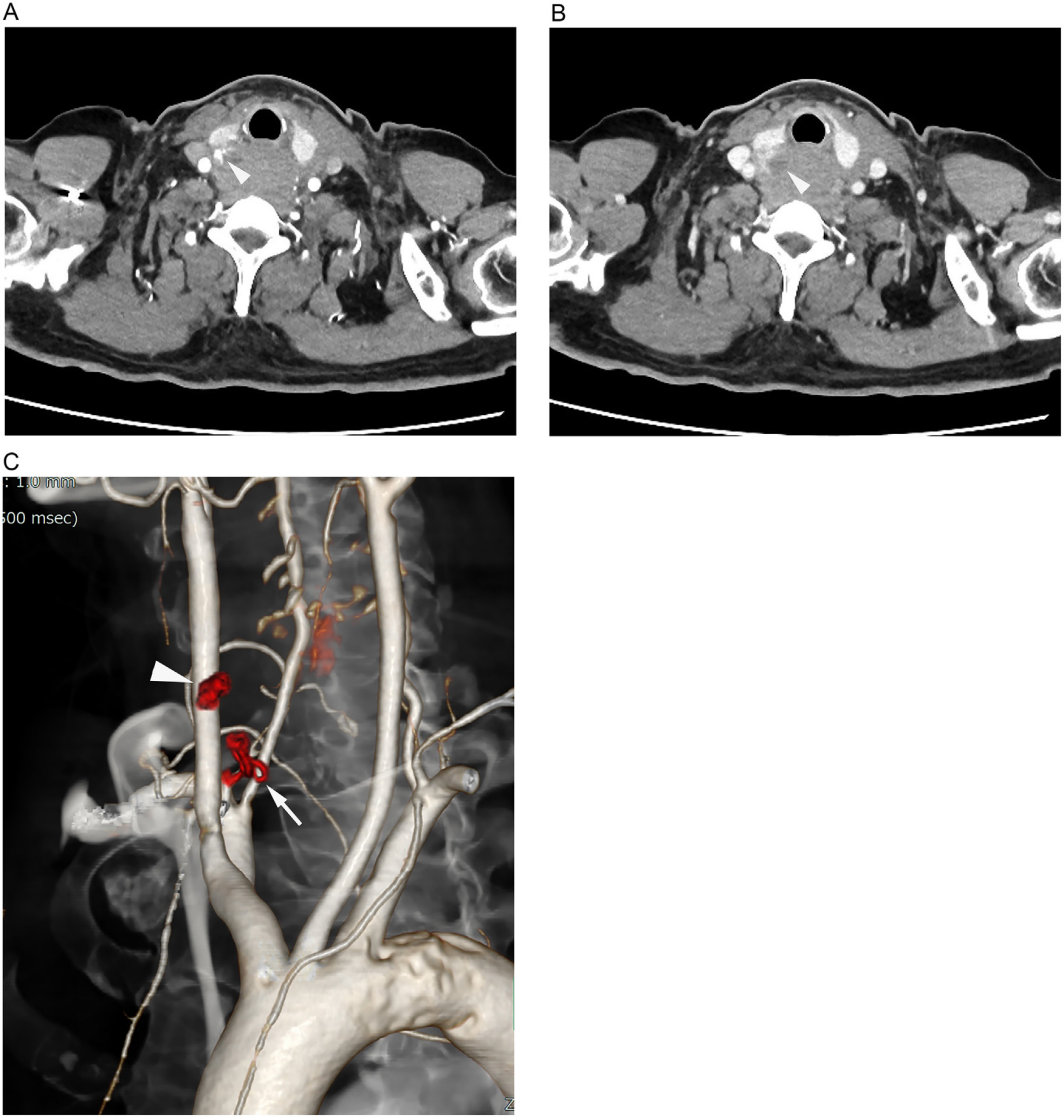
(A, B) Dynamic contrast-enhanced CT scans show extravasation of contrast media (arrowhead) posterior to the right lobe of the thyroid gland (white arrowhead), showing expansion. (C) A volume-rendering image shows the right inferior thyroid artery (white arrow) and the adjacent extravasation (white arrowhead) highlighting their proximity.

Initial precontrast CT showed medium- to high-density lesions extending from the neck to the mediastinum (Fig. 1A). The neck lesion was located dorsal to the right thyroid gland lobe (Fig. 1B), and in the mediastinum, it surrounded the esophagus (Fig. 1C, D). Bilateral hyperdense pleural effusions were also noted. Initially, an esophageal tumor was suspected, but cervical and mediastinal hematomas were later considered by the radiologists. Subsequent dynamic contrast-enhanced CT scans revealed contrast media extravasation dorsal to the right thyroid gland lobe (Fig. 2A), suggesting possible rupture of a parathyroid adenoma or the inferior thyroid artery. A hypodense nodule adjacent to the extravasation, presumed to be the right inferior parathyroid gland, did not show contrast enhancement (Fig. 2B). The nearby right inferior thyroid artery was suspected to be the source of the hemorrhage (Fig. 2C), leading to a diagnosis of extensive cervical and mediastinal hematoma caused by rupture of this artery.

**Fig. 3:**
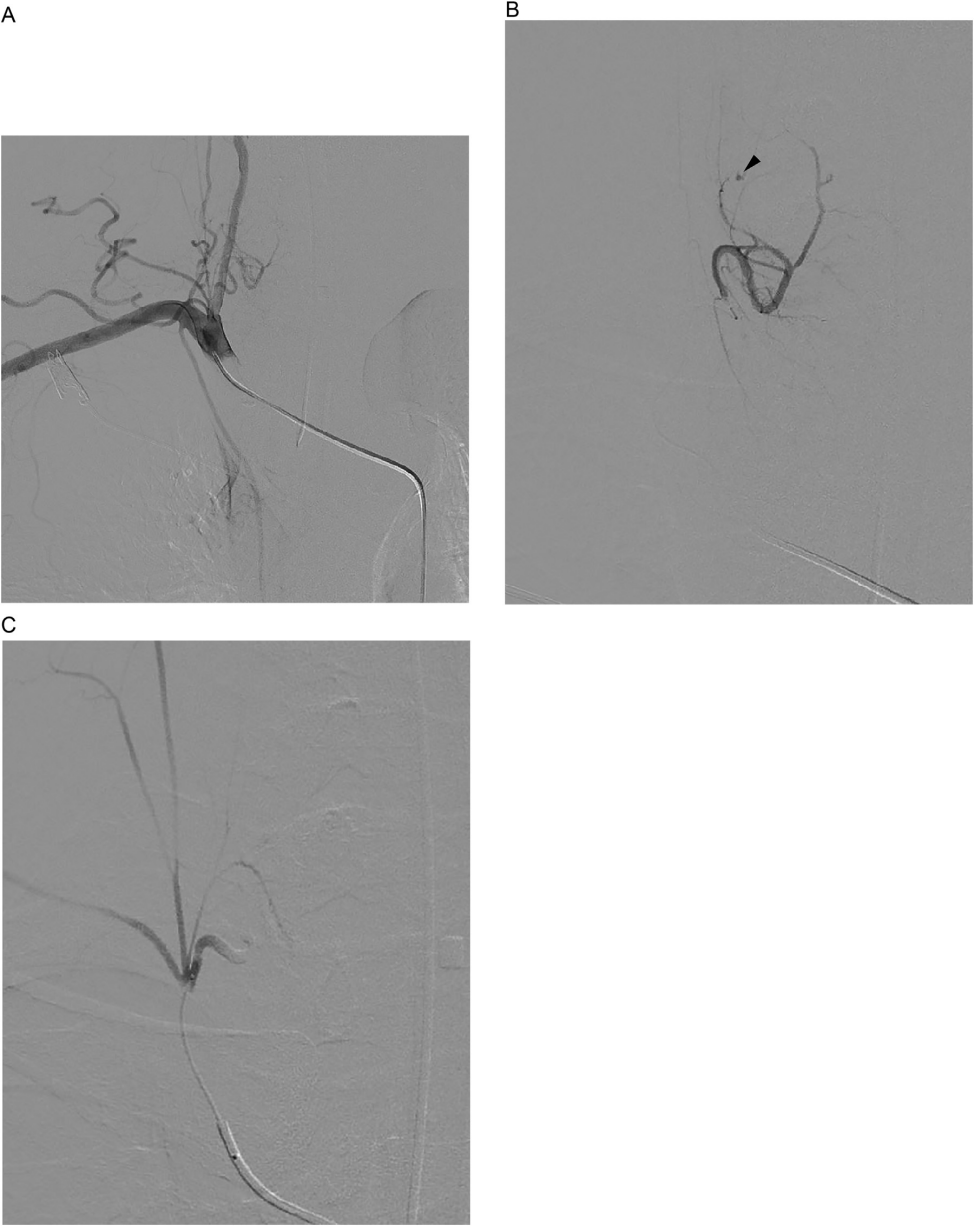
(A) An arteriogram of the right subclavian artery showing the right inferior thyroid artery branching off from the thyrocervical trunk. (B) An arteriogram of the right inferior thyroid artery depicts the site of extravasation. (C) An arteriogram of the thyrocervical trunk shows the occluded inferior thyroid artery.

The treatment plan involved removing the hematoma. Following endotracheal intubation, transarterial embolization (TAE) of the right inferior thyroid artery was performed. A 4Fr catheter was introduced into the right subclavian artery, and an angiogram confirmed extravasation from the right inferior thyroid artery (Fig. 3B). Then a 2.0 Fr microcatheter was advanced into this artery via the thyrocervical trunk, and a selective angiogram showed no communication with other arteries. The artery was embolized using a gelatin sponge (Fig. 3C). Following embolization, the cervical and mediastinal hematomas were surgically removed, and the right lower parathyroid gland was also resected, which showed no signs of adenoma.

## Discussion

Cervical hematoma arises from various causes. While trauma and postsurgical scenarios such as thyroidectomy and parathyroidectomy present straightforward diagnostic paths, spontaneous cervical hematomas have more complex etiologies [[1], [2], [3], [4], [5], [6], [7], [8]]. A similar complexity is seen in spontaneous mediastinal hematomas, which also encompass a range of causes [2,5,[9], [10], [11]], some of which may overlap with those of cervical hematomas. In this case, the hematoma extended into both the mediastinum and neck, necessitating a comprehensive evaluation to identify the responsible lesion.

The potential etiologies include ruptured thyroid arteries, arterial aneurysms, and artery dissection, with most reported cases involving ruptured aneurysms. For aneurysms in general, size may be a risk factor; however, this has not been definitively established. In one review, 2 patients with ruptured aneurysms had 4 cm aneurysms and 1 patient with hoarseness presented with a 5 cm aneurysm [12]. Five asymptomatic cases had aneurysms ranging from 2 to 6 cm [12]. Ruptures of nonaneurysmal thyroid arteries are rarer and their risk factors have not been investigated. Two reported cases had chronic asthma and hypertension complicated by cerebral infarction, respectively [1,2]. Our patient had hypertension, diabetes mellitus, chronic renal failure, and cerebral infarction. Diseases that compromise vascular integrity are considered potential risk factors for rupture of nonaneurysmal thyroid arteries. However, predicting such ruptures remains challenging.

The patient's hemodialysis treatment and associated heparin administration were not a cause but likely prevented effective hemostasis, resulting in extensive hematoma from the neck to the mediastinum. Previous cases have had spontaneous cervical and mediastinal hemorrhages linked to oral anticoagulant therapy and antiplatelet therapy [4,8,9]. Regarding spontaneous neck hematoma, all cases have resulted from excessive effects of warfarin [8], while spontaneous mediastinal hematomas have occurred with both normal and excessive effects of anticoagulant therapy and antiplatelet therapy [9]. We should remain aware that these therapies can promote spontaneous hematoma formation in the neck or mediastinum.

Dynamic contrast-enhanced CT was helpful in diagnosis, highlighting the extravasation of contrast media. This extravasation, located dorsal to the right thyroid gland lobe, pointed towards the spontaneous rupture of either a parathyroid adenoma or the inferior thyroid artery. An oval, hypodense structure resembling the right inferior parathyroid gland was observed; however, its lack of contrast enhancement in CT scans made a parathyroid adenoma less likely, as these typically show early phase contrast enhancement. Pathological examination confirmed the absence of a neoplastic component in the parathyroid gland. Close proximity of the inferior right thyroid artery to the site of extravasation raised suspicions of its rupture, a phenomenon reported both in aneurysmal [2,7,[12], [13], [14], [15]] and nonaneurysmal thyroid arteries [[1], [2], [3], [4]].

TAE of the thyroid artery effectively stopped the active bleeding. Various treatments for cervical hematomas, including TAE alone, surgery, or a combination of both, have been documented [1,3,12,[15], [16], [17]]. Given the extent of cervical and mediastinal hematoma in this patient, surgical removal with prior bleeding control via TAE was selected. Selective embolization of the thyroid artery is generally safe. Although branching from the vertebral artery has been noted [18], no connection with the spinal artery has been reported to date. In this case, TAE was performed without complications.

In conclusion, despite the varied etiologies of spontaneous cervical and mediastinal hematomas, bleeding from the thyroid artery should be considered a potential cause, enabling immediate diagnosis and prompt treatment.

## Patient consent

Written informed consent was obtained from the patient for publication of this case report. We are retaining the consent document or our own records.

## References

[1] Bageacu S, Prades JM, Kaczmarek D, Porcheron J (2005). Images in cardiothoracic surgery: spontaneous rupture of the inferior thyroid artery leading to life-threatening mediastinal hematoma. Ann Thorac Surg.

[2] Hoetzenecker K, Topker M, Klepetko W, Ankersmit HJ (2010). Spontaneous rupture of the inferior thyroid artery resulting in mediastinal hematoma. Interact Cardiovasc Thorac Surg.

[3] Stenner M, Helmstaedter V, Spuentrup E, Quante G, Huettenbrink KB (2010). Cervical hemorrhage due to spontaneous rupture of the superior thyroid artery: case report and review of the literature. Head Neck.

[4] Kieu V, Tassone P, Hobbs CG (2012). Successful conservative management of the spontaneous rupture of a superior thyroid artery aneurysm. ANZ J Surg.

[5] Ilyicheva E (2015). Spontaneous cervical-mediastinal hematoma caused by hemorrhage into parathyroid adenoma: a clinical case. Int J Surg Case Rep.

[6] Tessler I, Adi M, Diment J, Lahav Y, Halperin D, Cohen O (2020). Spontaneous neck hematoma secondary to parathyroid adenoma: a case series. Eur Arch Otorhinolaryngol.

[7] Rasteau S, Bonnet L, Jay-Caillierez L (2022). Spontaneous rupture of an aneurysm of the superior thyroid artery: a rare cause of acute respiratory failure. J Stomatol Oral Maxillofac Surg.

[8] Thomas SA, Moideen SP, George SM, Ipe S, Menon DG (2022). Management of spontaneous neck hematoma in patients on anticoagulant therapy: our experience from a tertiary care center. Indian J Otolaryngol Head Neck Surg.

[9] Mikubo M, Sonoda D, Yamazaki H (2017). Spontaneous non-traumatic mediastinal hematoma associated with oral anticoagulant therapy: a case report and literature review. Int J Surg Case Rep.

[10] Kumar KSS, Sharma P, Sherwani P, Dua R, Layek A (2022). Ruptured spontaneous bronchial artery pseudo aneurysm with large mediastinal hematoma and its interventional management: an acute chest emergency. Lung India.

[11] Aziz R, Khan A, Yousefi M, Khetani S, Choudhry H (2023). Spontaneous non-traumatic mediastinal hematoma in a patient on imatinib therapy for a gastrointestinal stromal tumor (GIST). Cureus.

[12] Garrett HE (2005). Heidepriem RW 3rd, Broadbent LP. Ruptured aneurysm of the inferior thyroid artery: repair with coil embolization. J Vasc Surg.

[13] Beal SL, Dublin AB, Stone WK (1987). Rupture of inferior thyroid artery aneurysm. J Vasc Surg.

[14] Watson DI, Benveniste GL, Sandhu AS, Raptis S, Stubberfield J (1994). Ruptured inferior thyroid aneurysm. Aust N Z J Surg.

[15] Terzi A, Pergher S, Falezza G, Calabrò F (2004). Cervical and mediastinal hematoma from ruptured aneurysm of the inferior thyroid artery. Eur J Cardiothorac Surg.

[16] Kos X, Henroteaux D, Dondelinger RF (2001). Embolization of a ruptured aneurysm of the inferior thyroid artery. Eur Radiol.

[17] Heckenkamp J, Aleksic M, Gawenda M, Krueger K, Reichert V, Brunkwall JS (2007). Endovascular treatment of a ruptured aneurysm of the inferior thyroid artery: case report and literature review. J Cardiovasc Surg (Torino).

[18] Weiglein AH (1996). A rare variant of thyroid gland vascularization. Surg Radiol Anat.

